# Sarcopenia is associated with insomnia in Japanese older adults: a cross-sectional study of data from the Nagasaki Islands study

**DOI:** 10.1186/s12877-020-01658-w

**Published:** 2020-07-28

**Authors:** Yuki Nagaura, Hideaki Kondo, Mako Nagayoshi, Takahiro Maeda

**Affiliations:** 1 Department of General Medicine, Nagasaki University Graduate School of Biomedical Sciences, Nagasaki, Japan; 2 International Institute for Integrative Sleep Medicine, University of Tsukuba, 1-2 Kasuga, Tsukuba, Ibaraki 305-8550 Japan; 3 Department of Community Medicine, Nagasaki University Graduate School of Biomedical Sciences, Nagasaki, Japan; 4 Department of Health Promotion, National Institute of Public Health, Wako, Japan

**Keywords:** Insomnia, Japanese, sarcopenia, Sleep duration

## Abstract

**Background:**

Sarcopenia is associated with increased mortality among older adults. Sleep-related problems have been studied as factors related to sarcopenia. This study was conducted to determine the relationship between sleep-related problems and sarcopenia among Japanese community-dwelling older adults using data from the Nagasaki Islands Study.

**Methods:**

This cross-sectional study analyzed data collected from 2017 to 2018. A total of 1592 older adults (575 men, 36.1%) aged 65 years or older participated. Sarcopenia was evaluated using the skeletal muscle mass index and grasp powers based on the criteria of the Asian Working Group for Sarcopenia. Odds ratios for sarcopenia were calculated using logistic regression analysis. Furthermore, subgroup analysis was performed based on the following tertiles of age: 65–70 years, 71–78 years, and 79–98 years.

**Results:**

The number of participants with sarcopenia was 238 (14.9%). The median age of participants in the sarcopenia group (80 years; interquartile range: 74–84) was significantly higher than in the non-sarcopenia group (73 years; interquartile range 69–79; *P* <  0.001). In the sarco*p*enia group, 70.9% of participants had difficulty initiating and/or maintaining sleep, sleep duration tended to be longer (*P* <  0.001), and 33.3% of participants’ sleep duration was over 9 h. In a logistic regression analysis for sarcopenia, advancing age was the most prominent factor, and the adjusted odds ratio (95% confidence interval) of facing difficulty initiating and/or maintaining sleep was 1.60 (1.14–2.25). Despite longer sleep duration being a significant factor in the univariable analysis, it was not significant in the multivariable analysis. In the logistic regression analysis for sarcopenia among older adults aged 79–98 years, the odds ratio (95% confidence interval) among women was significantly low at 0.53 (0.33–0.83).

**Conclusions:**

Sarcopenia is associated with difficulty initiating and/or maintaining sleep among Japanese older adults. In sarcopenia control measures, sleep/wake disorders related to insomnia are required to be evaluated in detail to help inform nursing and medical policy.

## Background

Sarcopenia is an age-related decline in skeletal muscle mass as well as muscle function. In a rapidly growing aging society, it is recognized as a significant factor for increased mortality [1]; thus, further clarification of the pathophysiology related to sarcopenia and the development of effective therapeutic interventions are needed in medical and nursing policy.

Sleep-related problems have been studied as factors related to sarcopenia. Some epidemiologic studies including older adults demonstrate that shorter and longer sleep time correlate with sarcopenia [2–6]. Additionally, decreased sleep quality among women, including prolonged sleep latency and day-time dysfunction, is associated with sarcopenia [7, 8]. Moreover, a tendency for delayed sleep phase and an eveningness chronotype are related to sarcopenia [7, 9].

In the common pathophysiological background of sarcopenia and sleep-related problems, the physiological relevance of myokine, a physiologically active substance derived from skeletal muscle, is important. A low level of irisin, one of the myokines, is associated with sarcopenia [10]. Fibronectin type III domain-containing protein 5, a precursor of irisin, is induced during exercise and cleaved and secreted from muscle as irisin. Irisin is involved in the synthesis of brain-derived neurotrophic factor (BDNF) in the hippocampus and contributes to memory improvement [11, 12]. Experimental evidence suggests that BDNF induces increased slow wave activity during sleep; cortical unilateral microinjection of BDNF induces higher slow wave activity in the injected hemisphere, compared to the contralateral one; and blocking the BDNF pathway suppresses local slow wave activity during sleep [13].

Further, the BDNF blood concentration of middle-aged adults with insomnia is lower than those of healthy adults, and the severity of insomnia is negatively correlated with BDNF value [14]. Although the role of myokine on the pathophysiology of sarcopenia remains unclear, it is hypothesized that the low level of irisin induces a decrease in BDNF synthesis and is related to the pathophysiology of insomnia in sarcopenia.

The results of previous basic research explain the pathophysiology of insomnia induced by sarcopenia; a mutual relationship between sarcopenia and insomnia is postulated to play a significant role in this condition. However, no studies refer to the relationship between the symptoms of insomnia and sarcopenia. Further, among Japanese community-dwelling older adults, the association between sleep-related problems and sarcopenia is unknown. This study was conducted to determine the relationship between sleep related problems and sarcopenia using data from the Nagasaki Islands Study (NaIS) performed in Goto City, where nearly 40% of adults are aged 65 years or greater.

## Methods

### Participants

**Fig. 1:**
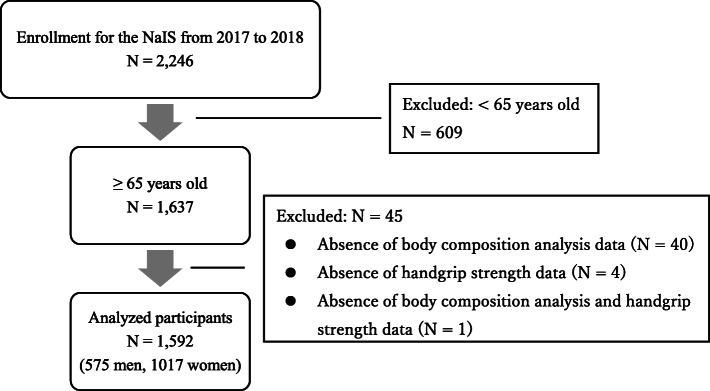
Flowchart showing how the analyzed participants were selected. *Abbreviations*: *NaIS* Nagasaki Islands Study

The NaIS is a prospective cohort study that has been performed since 2014 in Goto City in the Nagasaki prefecture, located on the western edge of Japan. The participants were recruited upon medical check-ups, and members of the general population aged 40 years or greater living in Goto City were targeted for enrollment. The recruitment process has been described elsewhere [15]. The present cross-sectional study was conducted in 2019 using data collected from May 2017 to June 2018, when the evaluation of sarcopenia was possible owing to the introduction of body composition analysis. Among the 2246 participants enrolled in the NaIS, the number of older adults aged 65 years or greater was 1637. A total of 45 participants were excluded from analysis in the current study. These included 40 participants whom body composition analysis was not performed, 4 whom grasp power was not performed, and 1 whom body composition analysis and grasp power were not performed. Consequently, a total of 1592 participants (575 men, 36.1%) were analyzed in the current study (Fig. 1). Approval for this study was granted by the Ethics Committee of Nagasaki University Graduate School of Biomedical Sciences (approval no. 14051404–8). Written informed consent was obtained from all participants.

### Survey and collection methods

Trained interviewers obtained information on the participants’ demographic and clinical characteristics, including sex, smoking status, drinking status, comorbidities, and care-related problems. Habitual drinkers were defined as people with a frequency of drinking ≥2 days per week and an alcohol intake ≥20 g of ethanol per day. Care-related problems were obtained in the following areas: staying at home all day, no outings, no hobbies, weakening of neighbor relations, weakening of human relations, falls, no long-distance walking, visual disturbance, stumbling, fear of falling, recent hospitalization, appetite loss, chewing difficulty, weight reduction, and muscle and fat wasting.

The symptoms of insomnia, including difficulty initiating sleep (DIS) and difficulty maintaining sleep (DMS), were assessed. Participants who had any of the symptoms were defined as having difficulty initiating and/or maintaining sleep (DIMS). Sleep duration was calculated from light-out time and get-up time.

Psychological distress was assessed using the Japanese version of the 6-item Kessler Psychological distress scale (K6). The score of each item ranges from 0 to 4, with global scores ranging from 0 to 24. The cut-off point for anxiety/emotional disorders screening was estimated at 4/5 with a sensitivity of 76–100% and specificity of 69–80% [16, 17].

A tendency toward malnutrition was defined as a body mass index (BMI) ≤ 20 kg/m^2^, for which a higher risk of mortality has been statistically observed in Japanese older adults [18]. Blood pressure at rest was measured using the cuff oscillometric method with a PASERA AVE-1500 (Shisei Datum, Tokyo, Japan). Individuals were considered as having hypertension if they had a systolic blood pressure of ≥140 mmHg and/or diastolic blood pressure of ≥90 mmHg, if they took blood pressure-lowering medications. Individuals were considered as having diabetes if their hemoglobin was A1c ≥ 6.5% or if they took blood glucose lowering medications. Individuals were considered as having dyslipidemia if their HDL-cholesterol was < 40 mg/dl or of if they took cholesterol- and/or triglyceride-lowering medications. The severity of chronic kidney disease was categorized into 5 categories based on the estimated glomerular filtration rate: G1–5 (G1 ≥ 90, G2 60–89, G3 30–59, G4 15–29, G5 < 15 mL/min/1.73m^2^) [19].

Measurement of handgrip strength was performed twice in each hand and mean strength was calculated. Skeletal muscle mass (SMM) was measured by using bioelectrical impedance analysis with an InBody 770 (InBody Japan, Tokyo, Japan). Skeletal muscle mass index (SMI) was calculated as follows: SMI = SMM/height^2^ (kg/m^2^) [20]. Sarcopenia was defined using the cut-off points of handgrip strength and SMI, but not gait speed, based on the criteria of the Asian Working Group for Sarcopenia (AWGS) as having both lower handgrip strength and lower SMI: handgrip strength (men < 26 kg; women < 18 kg) and SMI (men ≤7 kg/m^2^; women ≤5.7 kg/m^2^) [21].

### Statistical analysis

R version 3.5.2 (https://www.r-project.org/) and EZR version 1.40 (http://www.jichi.ac.jp/saitama-sct/SaitamaHP.files/statmed.html) were used for statistical analysis [22]. Continuous variables were presented as medians and interquartile ranges (IQR) where non-normally distributed. Comparisons of continuous variables were performed using Mann–Whitney U test. Categorical variables were presented as counts and percentages. Frequency analyses for categorical data were performed using Fisher’s exact test. The two-sided alpha level was set at 0.05.

Odds ratios (ORs) for sarcopenia were calculated via logistic regression analysis with sarcopenia as a dependent variable. The following were used as independent variables: age, sex, BMI, IHD, DIMS, sleep duration, and care related problems, including appetite loss, no hobbies, recent hospitalization, weakening of neighbor relations, visual disturbance, and without long-distance walking. Age was categorized based on the following quartiles: ≤ 68 years (reference group; ref), 69–73 years, 74–79 years, and ≥ 80 years. BMI was categorized into three groups: ≤ 20 kg/m^2^, 20–25 kg/m^2^ (ref), and > 25 kg/m^2^. Smoking was categorized into three groups: never (ref), current smoker, and ex-smoker. Sleep duration was categorized into 5 groups: < 6 h, 6–7 h, 7–8 h (ref), 8–9 h, and ≥ 9 h. The global score of K6 was categorized into two groups: < 5 (ref) and ≥ 5 (psychological distress group).

Sarcopenia and insomnia are prevalent with advancing age. Therefore, subgroup analysis was performed based on the following tertiles of age: 65–70 years, 71–78 years, and 79–98 years. In the second set logistic regression analyses for sarcopenia stratified by tertiles of age, the independent variables were sex, BMI, and DIMS.

## Results

Table 1 and Additional file 1 (Table S1) show the demographic and clinical characteristics. The number of participants with sarcopenia was 282 (17.7%). The median age (interquartile range) of participants with sarcopenia was significantly higher than that of participants without sarcopenia: 79 (74–84) years vs. 73 (68–78) years, respectively (*P* <  0.001). Among the sarcopenia group, a tendency toward spending a longer sleep duration was observed and the percentage of sleep duration ≥9 h was 32.0% (*P* <  0.001). Compared to the non-sarcopenia group, the percentages of DIS and DMS among participants in the sarcopenia group were significantly higher (*P* = 0.005 and *P* <  0.001, respectively), and the percentage of DISM in the sarcopenia group reached 69.0% (Table 2). Care-related problems in the sarcopenia group were prevalent; specifically, no hobbies, no long-distance walking (≥ 1 km), and appetite loss showed higher percentages (*P* <  0.001; Table 3). In the logistic regression analysis for sarcopenia, advancing age was the most prominent factor, and the adjusted OR (95% confidence interval; CI) of having DIMS was 1.56 (1.14–2.13). Moreover, longer sleep duration was a significant factor in the univariable analysis, but it was not significant in the multivariable analysis (Table 4).

**Table 1:** Demographic and sleep-related characteristics

N	1592
Sex, Men, n (%)	575 (36.1)
Age, median (IQR)	74 (69–80)
BMI kg/m^2^, median (IQR)	22.7 (20.6–24.9)
BMI ≤20 kg/m^2^, n (%)	319 (20.0)
BMI 20–25 kg/m^2^, n (%)	894 (56.2)
BMI > 25 kg/m^2^, n (%)	379 (23.8)
Habitual drinker, n (%)	38 (2.4)
Smoking status	
Never, n (%)	1117 (70.2)
Past, n (%)	369 (23.2)
Current, n (%)	106 (6.7)
Sleep duration	
< 6 h, n (%)	79 (5.0)
6–7 h, n (%)	216 (13.6)
7–8 h, n (%)	429 (27.0)
8–9 h, n (%)	482 (30.3)
≥ 9 h, n (%)	385 (24.2)
Insomnia symptoms	
DIS, n (%)	432 (27.2)
DMS, n (%)	592 (37.2)
DIMS, n (%)	844 (53.0)
K6 global score ≥ 5, n (%)	134 (8.4)

Abbreviations*: BMI* body mass index, *DIS* difficulty initiating sleep, *DIMS* difficulty initiating and/or maintaining sleep, *DMS* difficulty maintaining sleep, *IQR* interquartile range

**Table 2:** Comparison of demographic and sleep-related characteristics between non-sarcopenia group and sarcopenia group

	Non-sarcopenia	Sarcopenia	*P* value
N	1310	282	
Male, n (%)	482 (36.8)	93 (33.0)	0.25
Age years, median (IQR)	73.0 (68.0–78.0)	79.0 (74.0–84.0)	< 0.001
65–68, n (%)	431 (32.9)	26 (9.2)	< 0.001
69–73, n (%)	326 (24.9)	56 (19.9)	
74–79, n (%)	332 (25.3)	80 (28.4)	
> 80, n (%)	221 (16.9)	120 (42.6)	
BMI kg/m^2^, median (IQR)	23.1 (21.0–25.2)	20.7 (19.0–22.7)	< 0.001
BMI ≤20 kg/m^2^, n (%)	208 (15.9)	111 (39.4)	< 0.001
BMI 20–25 kg/m^2^, n (%)	753 (57.5)	141 (50.0)	
BMI > 25 kg/m^2^, n (%)	349 (26.6)	30 (10.6)	
Habitual drinker, n (%)	35 (2.7)	3 (1.1)	0.13
Smoking status			
Never, n (%)	909 (69.4)	208 (73.8)	0.29
Past, n (%)	309 (23.6)	60 (21.3)	
Current, n (%)	92 (7.0)	14 (5.0)	
Sleep duration			
< 6 h, n (%)	70 (5.3)	9 (3.2)	< 0.001
6–7 h, n (%)	190 (14.5)	26 (9.3)	
7–8 h, n (%)	366 (27.9)	63 (22.4)	
8–9 h, n (%)	389 (29.7)	93 (33.1)	
≥ 9 h, n (%)	295 (22.5)	90 (32.0)	
Insomnia symptoms			
DIS, n (%)	336 (25.7)	96 (34.2)	0.005
DMS, n (%)	452 (34.5)	140 (49.6)	< 0.001
DIMS, n (%)	650 (49.6)	194 (69.0)	< 0.001
K6 global score ≥ 5, n (%)	102 (7.8)	32 (11.3)	0.06

Abbreviations: *BMI* body mass index, *DIS* difficulty initiating sleep, *DIMS* difficulty initiating and/or maintaining sleep, *DMS* difficulty maintaining sleep, *IQR* interquartile range

**Table 3:** Comparison of care-related problems and clinical characteristics between non-sarcopenia group and sarcopenia group

	Non-sarcopenia	Sarcopenia	*P* value
N	1310	282	
Care-related problems			
Staying at home all day, n (%)	151 (11.5)	57 (20.2)	< 0.001
No outing, n (%)	52 (4.0)	25 (8.9)	0.001
No hobbies, n (%)	189 (14.4)	67 (23.8)	< 0.001
Weakening of neighbor relations, n (%)	219 (16.7)	66 (23.4)	0.01
Weakening of human relations, n (%)	132 (10.1)	40 (14.2)	0.056
Falls, n (%)	218 (16.7)	59 (20.9)	0.1
No long-distance walking, n (%)	213 (16.3)	90 (31.9)	< 0.001
Visual disturbance, n (%)	33 (2.5)	15 (5.3)	0.02
Stumbling, n (%)	162 (12.4)	42 (14.9)	0.28
Fear of falling, n (%)	10 (0.8)	10 (3.5)	0.001
Recent hospitalization, n (%)	136 (10.4)	46 (16.3)	0.007
Appetite loss, n (%)	32 (2.4)	22 (7.8)	< 0.001
Chewing difficulty, n (%)	55 (4.2)	18 (6.4)	0.12
Weight reduction, n (%)	85 (6.5)	30 (10.7)	0.02
Muscle and fat wasting, n (%)	252 (19.2)	78 (27.7)	0.002
Hypertension, n (%)	803 (61.3)	182 (64.5)	0.34
Diabetes, n (%)	361 (27.6)	64 (22.7)	0.1
Dyslipidemia, n (%)	372 (28.4)	72 (25.5)	0.34
Ischemic heart disease, n (%)	98 (7.6)	38 (13.6)	0.002
Stroke, n (%)	69 (5.4)	19 (6.8)	0.39
Chronic kidney disease			
G1, n (%)	87 (6.6)	31 (11.0)	0.005
G2, n (%)	852 (65.0)	156 (55.3)	
G3, n (%)	366 (27.9)	95 (33.7)	
G4–5, n (%)	5 (0.4)	0 (0.0)

**Table 4:** Logistic regression analysis for sarcopenia as a dependent variable

Independent variable	Univariable analysis Odds ratio (95% CI)	Multivariable analysis Odds ratio (95%CI)
Age		
65–68(reference)	1	1
69–73	2.85 (1.75–4.63)	3.13 (1.86–5.28)
74–79	3.99 (2.51–6.36)	3.72 (2.23–6.20)
> 80	9.00 (5.72–14.20)	7.79 (4.63–13.10)
Female (vs. Male)	1.18 (0.90–1.55)	1.11 (0.81–1.51)
BMI		
≤20 kg/m2	2.85 (2.13–3.82)	3.33 (2.39–4.63)
20–25 kg/m2(reference)	1	1
> 25 kg/m2	0.46 (0.30–0.70)	0.46 (0.29–0.71)
IHD (vs. non-IHD)	1.91 (1.28–2.84)	1.49 (0.95–2.34)
Care-related problems		
Appetite loss	3.37 (1.93–5.90)	2.04 (1.04–4.01)
No hobbies	1.85 (1.35–2.53)	1.77 (1.21–2.57)
Recent hospitalization	1.68 (1.17–2.42)	1.40 (0.92–2.12)
Weakening of neighbor relations	1.52 (1.11–2.08)	1.27 (0.88–1.84)
Visual disturbance	2.18 (1.17–4.07)	1.20 (0.59–2.43)
Without long-distance walking	2.41 (1.81–3.23)	1.39 (0.97–2.00)
DIMS (vs. non-DIMS)	2.26 (1.72–2.98)	1.56 (1.14–2.13)
Sleep duration		
< 6 h	0.75 (0.36–1.57)	0.95 (0.42–2.14)
6–7 h	0.80 (0.49–1.30)	0.94 (0.55–1.62)
7–8 h	1	1
8–9 h	1.39 (0.98–1.97)	1.11 (0.75–1.64)
≥ 9 h	1.77 (1.24–2.53)	1.21 (0.81–1.81)

Abbreviations*: BMI* body mass index, *CI* confidence interval, *DIS* difficulty initiating sleep, *DIMS* difficulty initiating and/or maintaining sleep, *DMS* difficulty maintaining sleep, *IHD* ischemic heart disease, *IQR* interquartile range

**Fig. 2:**
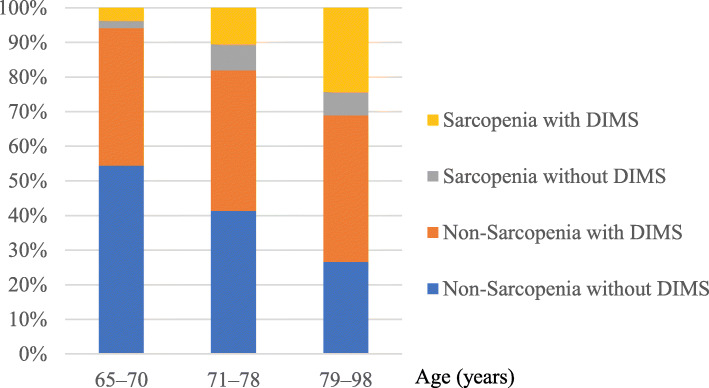
Percentage of sarcopenia and DIMS stratified by tertile of age. *Abbreviations*: *DIMS* difficulty initiating and/or maintaining sleep

In the subgroup analysis stratified by tertile of age, the numbers of participants with sarcopenia among older adults aged 65–70, 71–78, and 79–98 years were 32 (5.8%), 103 (18.0%) and 147 (31.2%; Additional file 1: Table S2–4), respectively. The percentages of sarcopenia with DIMS among older adults aged 65–70, 71–78, and 79–98 years were 3.6, 10.5, and 24.3%, respectively (Fig. 2). The ORs (95% CI) of having DIMS among older adults aged 65–70, 71–78, and 79–98 years for sarcopenia were 2.25 (1.06–4.75), 1.48 (0.93–2.34), and 2.07 (1.28–3.33), respectively (Table 5).

**Table 5:** Logistic regression analysis, stratified by tertile of age, for sarcopenia as a dependent variable

Independent variable	Multivariable analysis Odds ratio (95%CI)
65–70 years	
Women (vs. Men)	0.86 (0.38–1.94)
BMI (increment of 1 kg/m^2^)	0.80 (0.70–0.90)
DIMS (vs. non-DIMS)	2.25 (1.06–4.75)
71–78 years	
Women (vs. Men)	1.61 (0.97–2.70)
BMI (increment of 1 kg/m^2^)	0.76 (0.70–0.83)
DIMS (vs. non-DIMS)	1.48 (0.93–2.34)
79–98 years	
Women (vs. Men)	0.79 (0.51–1.22)
BMI (increment of 1 kg/m^2^)	0.75 (0.69–0.82)
DISM (vs. non-DISM)	2.07 (1.28–3.33)

Abbreviations: *BMI* body mass index, *DIMS* difficulty initiating and/or maintaining sleep

## Discussion

This study is the first to examine the relationship between sarcopenia and insomnia among Japanese community-dwelling older adults. Some previous studies have focused on the relationship between sarcopenia and sleep duration. In the present multivariable analysis, however, DIMS was a significant factor, rather than sleep duration. Although decreased subjective sleep quality, prolonged sleep latency, and tendency of delayed sleep phase are reported to be associated with sarcopenia [7–9], there is no study that specifically explores DIMS and sarcopenia.

In the present study, there were few participants with a short sleep duration. Only 22 participants (1.4%) had a sleep duration < 5 h, and 79 participants (5%) had a sleep duration < 6 h. The relationship between short sleep duration and sarcopenia was reported in three previous studies [2, 3, 5]. Two of the studies examined the relationship among adults aged 40 years or greater, and the participants were not restricted to older adults [2, 3]. Therefore, more large-scale examination is required to determine the relationship between short sleep duration and sarcopenia among older adults.

In the present study, although a sleep duration ≥9 h was associated with sarcopenia in univariable analysis, long sleep duration was not a significant factor in multivariable analysis. Long sleep duration is reported to be associated with sarcopenia [2–6]. In an epidemiological study without using polysomnography, sleep duration is not necessarily the actual sleep duration. Particular attention was given to participants with insomnia who spent a long time in bed. In older adults, lower sleep efficiency is frequently observed as a result of increased wake time after sleep onset [23]. In a study in which participants’ mean age (± standard deviation; SD) was 41.3 ± 18.4 years, self-reported habitual mean (± SD) sleep duration was 7.6 ± 1.6 h, and mean (± SD) total sleep time obtained by polysomnography was 6.2 ± 1.6 h [24]. In the present study, the percentage of participants with DIMS, especially DMS, increased with a longer sleep duration (Additional file 1: Table S5).

In the general Japanese adult population, the prevalence of DIMS in men and women is reported as 17.3 and 21.5%, respectively. The prevalence increases with advancing age: 60–69, 70–79, and ≥ 80 years; 21.9, 20.5, and 30.5% in men; 20.3, 26.3, and 40.3% in women, respectively [25]. In this previous report, DIMS was defined as difficulty initiating sleep within 30 min three or more times a week and/or difficulty maintaining sleep three or more times a week in the last month. In the present study, DIMS was more prevalent than in that previous study, because frequency of insomnia symptoms were not included in the definition of DIMS.

The associations between sarcopenia and DIMS were observed among older adults aged 65–70 and 79–98 years, but not those aged 71–78 years. The increase of sarcopenia surpassed the increase of DISM in the 71–78 years group. In the 65–70 years group, the prevalence of sarcopenia was fewer and the 95% CI of OR for sarcopenia was wider than the other groups. To be adjust for confounding factors, a larger scale study is required for this group.

In previous studies, insomnia symptoms have not been considered in the evaluation of sleep problems related to sarcopenia among older adults. Future studies should investigate in detail not only chronic insomnia disorder, but also other sleep/wake disorders related to insomnia symptoms, including restless legs syndrome/Willis-Ekbom disease.

### Study limitations

The present study possesses some limitations of note. Gait speed was not assessed in the present study. The evaluation of physical performance is an important factor to diagnose sarcopenia. Low gait speed and/or low handgrip strength were included in AWGS 2014 criteria. Therefore, participants whose gait speed and muscle mass were low, but handgrip strength was within normal range, were classified as the “Non-sarcopenia” group. In AWGS 2019 criteria, either calf circumference (CC) or the SARC-F or SARC-CalF questionnaires is recommended for case-finding, and either 5-time chair stand test or 6-m walk or short physical performance battery is recommended for assessment of physical performance [26]. In the present study, these items were not assessed. Further studies are required based on the AWGS 2019 criteria.

In the present data collection using questionnaires by interviewers, actual total sleep duration could not be confirmed; thus, an objective evaluation using polysomnography is needed to measure total sleep time accurately. Additionally, the severity of insomnia symptoms was not considered. Sleep/wake disorders related to insomnia, including chronic insomnia disorder, sleep disordered breathing, circadian rhythm sleep/wake disorders, restless legs syndrome/Willis-Ekbom disease, and rapid eye movement sleep behavior disorder were not examined systematically.

Finally, although the relationship between sarcopenia and DIMS was observed, it remains unknown whether insomnia induces sarcopenia in the present cross-sectional study. To confirm the effects of insomnia on developing sarcopenia, it is necessary to conduct large prospective cohort studies and longitudinal studies targeting the patients with insomnia.

## Conclusions

Sarcopenia was found to be associated with difficulty initiating and/or maintaining sleep among Japanese community-dwelling older adults. Consequently, in sarcopenia control measures, sleep/wake disorders related to insomnia must be evaluated in detail. Further, basic and clinical studies are needed to elucidate the link between sleep/wake disorders and sarcopenia.

## Supplementary information

**Additional file 1: Table S1.** Care-related problems and clinical characteristics. **Table S2–1.** Comparison of demographic and sleep-related characteristics between non-sarcopenia group and sarcopenia group aged 65–70 years. **Table S2–2.** Comparison of care-related problems and clinical characteristics between non-sarcopenia group and sarcopenia group aged 65–70 years. **Table S3–1.** Comparison of demographic and sleep-related characteristics between non-sarcopenia group and sarcopenia group aged 71–78 years. **Table S3–2.** Comparison of care-related problems and clinical characteristics between non-sarcopenia group and sarcopenia group aged 71–78 years. **Table S4–1.** Comparison of demographic and sleep-related characteristics between non-sarcopenia group and sarcopenia group aged 79–98 years. **Table S4–2.** Comparison of care-related problems and clinical characteristics between non-sarcopenia group and sarcopenia group aged 79–98 years. **Table S5.** Symptoms of insomnia and sleep duration.

## Data Availability

The dataset used and/or analyzed during the current study is available from the corresponding author on reasonable request.

## Abbreviations

AWGS: Asian Working Group for Sarcopenia
BDNF: Brain-derived neurotrophic factor
BMI: Body mass index
CC: Calf circumference
CI: Confidence interval
DIS: Difficulty initiating sleep
DIMS: Difficulty initiating and/or maintaining sleep
DMS: Difficulty maintaining sleep
IHD: Ischemic heart disease
IQR: Interquartile range
K6: Kessler Psychological distress scale
NaIS: Nagasaki Islands Study
OR: Odds ratio
SMI: Skeletal muscle mass index
SMM: Skeletal muscle mass
