# Supplementary material for: Sarcopenia is associated with insomnia in Japanese older adults: a cross-sectional study of data from the Nagasaki Islands study
Source: BMC Geriatr. 2020 Jul 28;20:256. doi: 10.1186/s12877-020-01658-w (PMC7388230; doi:10.1186/s12877-020-01658-w)
Supplement: Supplementary file 1 — Additional file 1: Table S1. Care-related problems and clinical characteristics. Table S2–1. Comparison of demographic and sleep-related characteristics between non-sarcopenia group and sarcopenia group aged 65–70 years. Table S2–2. Comparison of care-related problems and clinical characteristics between non-sarcopenia group and sarcopenia group aged 65–70 years. Table S3–1. Comparison of demographic and sleep-related characteristics between non-sarcopenia group and sarcopenia group aged 71–78 years. Table S3–2. Comparison of care-related problems and clinical characteristics between non-sarcopenia group and sarcopenia group aged 71–78 years. Table S4–1. Comparison of demographic and sleep-related characteristics between non-sarcopenia group and sarcopenia group aged 79–98 years. Table S4–2. Comparison of care-related problems and clinical characteristics between non-sarcopenia group and sarcopenia group aged 79–98 years. Table S5. Symptoms of insomnia and sleep duration. [file 12877_2020_1658_MOESM1_ESM.docx]

Table S1．Care-related problems and clinical characteristics

| N | 1592 |
| --- | --- |
| Care-related problems |  |
| Staying at home all day, n (%) | 208 (13.1) |
| No outing, n (%) | 77 (4.8) |
| No hobbies, n (%) | 256 (16.1) |
| Weakening of neighbor relations, n (%) | 285 (17.9) |
| Weakening of human relations, n (%) | 172 (10.8) |
| Falls, n (%) | 277 (17.5) |
| No long-distance walking, n (%) | 303 (19.0) |
| Visual disturbance, n (%) | 48 (3.0) |
| Stumbling, n (%) | 204 (12.8) |
| Fear of falling, n (%) | 20 (1.3) |
| Recent hospitalization, n (%) | 182 (11.4) |
| Appetite loss, n (%) | 54 (3.4) |
| Chewing difficulty, n (%) | 73 (4.6) |
| Weight reduction, n (%) | 115 (7.2) |
| Muscle and fat wasting, n (%) | 330 (20.7) |
| Hypertension, n (%) | 985 (61.9) |
| Diabetes, n (%) | 425 (26.7) |
| Dyslipidemia, n (%) | 444 (27.9) |
| Ischemic heart disease, n (%) | 136 (8.7) |
| Stroke, n (%) | 88 (5.6) |
| Chronic kidney disease |  |
| G1, n (%) | 118 (7.4) |
| G2, n (%) | 1008 (63.3) |
| G3, n (%) | 461 (29.0) |
| G4–5, n (%) | 5 (0.3) |

Table S2-1. Comparison of demographic and sleep-related characteristics between non-sarcopenia group and sarcopenia group aged 65-70 years

|  | Non-sarcopenia | Sarcopenia | *P* value |
| --- | --- | --- | --- |
| N | 518 | 32 |  |
| Male, n (%) | 179 (34.6) | 10 (31.2) | 0.85 |
| Age years, median (IQR) | 68.0 (66.0–69.0) | 68.0 (67.0–69.0) | 0.26 |
| BMI kg/m^2^, median (IQR) | 23.1 (20.8–25.1) | 20.9 (18.9–23.3) | 0.001 |
| BMI ≤20 kg/m^2^, n (%) | 94 (18.1) | 12 (37.5) | 0.04 |
| BMI 20–25 kg/m^2^, n (%) | 291 (56.2) | 15 (46.9) |  |
| BMI >25 kg/m^2^, n (%) | 133 (25.7) | 5 (15.6) |  |
| Habitual drinker, n (%) | 19 (3.7) | 1 (3.1) | 1 |
| Smoking status |  |  |  |
| Never, n (%) | 352 (68.0) | 18 (56.2) | 0.25 |
| Past, n (%) | 114 (22.0) | 11 (34.4) |  |
| Current, n (%) | 52 (10.0) | 3 (9.4) |  |
| Sleep duration |  |  |  |
| <6 hours, n (%) | 45 (8.7) | 4 (12.9) | 0.55 |
| 6–7 hours, n (%) | 90 (17.4) | 7 (22.6) |  |
| 7–8 hours, n (%) | 164 (31.7) | 7 (22.6) |  |
| 8–9 hours, n (%) | 130 (25.1) | 6 (19.4) |  |
| Insomnia symptoms | 89 (17.2) | 7 (22.6) |  |
| DIS, n (%) | 123 (23.7) | 10 (31.2) | 0.39 |
| DMS, n (%) | 132 (25.5) | 12 (37.5) | 0.15 |
| DIMS, n (%) | 219 (42.3) | 20 (62.5) | 0.03 |
| K6 global score ≥5, n (%) | 40 (7.7) | 2 (6.2) | 1 |

*Abbreviations:* *BMI* body mass index; *DIS* difficulty initiating sleep; *DIMS* difficulty initiating and/or maintaining sleep; *DMS* difficulty maintaining sleep; *IQR* interquartile range.

Table S2-2. Comparison of care-related problems and clinical characteristics between non-sarcopenia group and sarcopenia group aged 65-70 years

|  | Non-sarcopenia | Sarcopenia | *P* value |
| --- | --- | --- | --- |
| N | 518 | 32 |  |
| Care-related problems |  |  |  |
| Staying at home all day, n (%) | 41 (7.9) | 3 (9.4) | 0.74 |
| No outing, n (%) | 15 (2.9) | 1 (3.1) | 1 |
| No hobbies, n (%) | 83 (16.0) | 8 (25.0) | 0.22 |
| Weakening of neighbor relations, n (%) | 99 (19.1) | 5 (15.6) | 0.82 |
| Weakening of human relations, n (%) | 50 (9.7) | 3 (9.4) | 1 |
| Falls, n (%) | 71 (13.8) | 8 (25.0) | 0.11 |
| No long-distance walking, n (%) | 29 (5.6) | 2 (6.2) | 0.7 |
| Visual disturbance, n (%) | 9 (1.7) | 1 (3.1) | 0.45 |
| Stumbling, n (%) | 54 (10.4) | 5 (15.6) | 0.37 |
| Fear of falling, n (%) | 0 (0.0) | 1 (3.1) | 0.06 |
| Recent hospitalization, n (%) | 45 (8.7) | 3 (9.4) | 0.75 |
| Appetite loss, n (%) | 12 (2.3) | 2 (6.2) | 0.19 |
| Chewing difficulty, n (%) | 14 (2.7) | 0 (0.0) | 1 |
| Weight reduction, n (%) | 34 (6.6) | 4 (12.5) | 0.27 |
| Muscle and fat wasting, n (%) | 87 (16.8) | 7 (21.9) | 0.47 |
| Hypertension, n (%) | 266 (51.4) | 15 (46.9) | 0.72 |
| Diabetes, n (%) | 139 (26.8) | 7 (21.9) | 0.68 |
| Dyslipidemia, n (%) | 136 (26.3) | 11 (34.4) | 0.31 |
| Ischemic heart disease, n (%) | 25 (5.0) | 1 (3.2) | 1 |
| Stroke, n (%) | 21 (4.2) | 2 (6.5) | 0.64 |
| Chronic kidney disease |  |  |  |
| G1, n (%) | 52 (10.0) | 5 (15.6) | 0.24 |
| G2, n (%) | 379 (73.2) | 19 (59.4) |  |
| G3, n (%) | 86 (16.6) | 8 (25.0) |  |
| G4–5, n (%) | 1 (0.2) | 0 (0.0) |  |

Table S3-1. Comparison of demographic and sleep-related characteristics between non-sarcopenia group and sarcopenia group aged 71-78 years

|  | Non-sarcopenia | Sarcopenia | *P* value |
| --- | --- | --- | --- |
| N | 468 | 103 |  |
| Male, n (%) | 186 (39.7) | 26 (25.2) | 0.007 |
| Age years, median (IQR) | 74.0 (72.0–77.0) | 75.0 (73.0–77.0) | 0.4 |
| BMI kg/m^2^, median (IQR) | 23.1 (21.0–25.3) | 20.1 (18.5–22.2) | < 0.001 |
| BMI ≤20 kg/m^2^, n (%) | 74 (15.8) | 50 (48.5) | < 0.001 |
| BMI 20–25 kg/m^2^, n (%) | 258 (55.1) | 41 (39.8) |  |
| BMI >25 kg/m^2^, n (%) | 136 (29.1) | 12 (11.7) |  |
| Habitual drinker, n (%) | 15 (3.2) | 2 (1.9) | 0.75 |
| Smoking status |  |  |  |
| Never, n (%) | 324 (69.2) | 79 (76.7) | 0.18 |
| Past, n (%) | 112 (23.9) | 16 (15.5) |  |
| Current, n (%) | 32 (6.8) | 8 (7.8) |  |
| Sleep duration |  |  |  |
| <6 hours, n (%) | 19 (4.1) | 3 (2.9) | 0.13 |
| 6–7 hours, n (%) | 82 (17.5) | 10 (9.7) |  |
| 7–8 hours, n (%) | 133 (28.4) | 25 (24.3) |  |
| 8–9 hours, n (%) | 143 (30.6) | 37 (35.9) |  |
| ≥9 hours, n (%) | 91 (19.4) | 28 (27.2) |  |
| Insomnia symptoms |  |  |  |
| DIS, n (%) | 122 (26.1) | 30 (29.1) | 0.54 |
| DMS, n (%) | 157 (33.5) | 44 (42.7) | 0.09 |
| DIMS, n (%) | 232 (49.6) | 60 (58.3) | 0.13 |
| K6 global score ≥5, n (%) | 37 (7.9) | 9 (8.7) | 0.84 |

*Abbreviations:* *BMI* body mass index; *DIS* difficulty initiating sleep; *DIMS* difficulty initiating and/or maintaining sleep; *DMS* difficulty maintaining sleep; *IQR* interquartile range.

Table S3-2. Comparison of care-related problems and clinical characteristics between non-sarcopenia group and sarcopenia group aged 71-78 years

|  | Non-sarcopenia | Sarcopenia | *P* value |
| --- | --- | --- | --- |
| N | 468 | 103 |  |
| Care-related problems |  |  |  |
| Staying at home all day, n (%) | 66 (14.1) | 17 (16.5) | 0.54 |
| No outing, n (%) | 18 (3.8) | 3 (2.9) | 1 |
| No hobbies, n (%) | 69 (14.8) | 28 (27.2) | 0.004 |
| Weakening of neighbor relations, n (%) | 67 (14.3) | 22 (21.4) | 0.1 |
| Weakening of human relations, n (%) | 43 (9.2) | 16 (15.5) | 0.07 |
| Falls, n (%) | 86 (18.4) | 19 (18.4) | 1 |
| No long-distance walking, n (%) | 70 (15.0) | 25 (24.3) | 0.03 |
| Visual disturbance, n (%) | 11 (2.4) | 5 (4.9) | 0.18 |
| Stumbling, n (%) | 62 (13.2) | 10 (9.7) | 0.41 |
| Fear of falling, n (%) | 4 (0.9) | 0 (0.0) | 1 |
| Recent hospitalization, n (%) | 50 (10.7) | 16 (15.5) | 0.17 |
| Appetite loss, n (%) | 10 (2.1) | 7 (6.8) | 0.02 |
| Chewing difficulty, n (%) | 17 (3.6) | 4 (3.9) | 0.78 |
| Weight reduction, n (%) | 23 (4.9) | 8 (7.8) | 0.23 |
| Muscle and fat wasting, n (%) | 94 (20.1) | 27 (26.2) | 0.18 |
| Hypertension, n (%) | 303 (64.7) | 58 (56.3) | 0.12 |
| Diabetes, n (%) | 130 (27.8) | 23 (22.3) | 0.27 |
| Dyslipidemia, n (%) | 134 (28.6) | 25 (24.3) | 0.4 |
| Ischemic heart disease, n (%) | 33 (7.1) | 11 (10.8) | 0.22 |
| Stroke, n (%) | 25 (5.4) | 4 (3.9) | 0.8 |
| Chronic kidney disease |  |  |  |
| G1, n (%) | 28 (6.0) | 19 (18.4) | 0.001 |
| G2, n (%) | 312 (66.7) | 61 (59.2) |  |
| G3, n (%) | 128 (27.4) | 23 (22.3) |  |
| G4–5, n (%) | 0 (0.0) | 0 (0.0) |  |

Table S4-1. Comparison of demographic and sleep-related characteristics between non-sarcopenia group and sarcopenia group aged 79-98 years

|  | Non-sarcopenia | Sarcopenia | *P* value |
| --- | --- | --- | --- |
| N | 324 | 147 |  |
| Male, n (%) | 117 (36.1) | 57 (38.8) | 0.61 |
| Age years, median (IQR) | 82.0 (80.0–84.0) | 84.0 (81.0–86.5) | < 0.001 |
| BMI kg/m^2^, median (IQR) | 23.2 (21.3–25.0) | 20.9 (19.5–22.7) | < 0.001 |
| BMI ≤20 kg/m^2^, n (%) | 40 (12.3) | 49 (33.3) | < 0.001 |
| BMI 20–25 kg/m^2^, n (%) | 204 (63.0) | 85 (57.8) |  |
| BMI >25 kg/m^2^, n (%) | 80 (24.7) | 13 (8.8) |  |
| Habitual drinker, n (%) | 1 (0.3) | 0 (0.0) | 1 |
| Smoking status |  |  |  |
| Never, n (%) | 233 (71.9) | 111 (75.5) | 0.77 |
| Past, n (%) | 83 (25.6) | 33 (22.4) |  |
| Current, n (%) | 8 (2.5) | 3 (2.0) |  |
| Sleep duration |  |  |  |
| <6 hours, n (%) | 6 1.9) | 2 (1.4) | 0.99 |
| 6–7 hours, n (%) | 18 (5.6) | 9 (6.1) |  |
| 7–8 hours, n (%) | 69 (21.3) | 31 (21.1) |  |
| 8–9 hours, n (%) | 116 (35.8) | 50 (34.0) |  |
| ≥9 hours, n (%) | 115 (35.5) | 55 (37.4) |  |
| Insomnia symptoms |  |  |  |
| DIS, n (%) | 91 (28.2) | 56 (38.4) | 0.03 |
| DMS, n (%) | 163 (50.5) | 84 (57.1) | 0.2 |
| DIMS, n (%) | 199 (61.4) | 114 (78.1) | < 0.001 |
| K6 global score ≥5, n (%) | 102 (7.8) | 32 (11.3) | 0.06 |

*Abbreviations:* *BMI* body mass index; *DIS* difficulty initiating sleep; *DIMS* difficulty initiating and/or maintaining sleep; *DMS* difficulty maintaining sleep; *IQR* interquartile range.

Table S4-2. Comparison of care-related problems and clinical characteristics between non-sarcopenia group and sarcopenia group aged 79-98 years

|  | Non-sarcopenia | Sarcopenia | *P* value |
| --- | --- | --- | --- |
| N | 324 | 147 |  |
| Care-related problems |  |  |  |
| Staying at home all day, n (%) | 44 (13.6) | 37 (25.2) | 0.004 |
| No outing, n (%) | 19 (5.9) | 21 (14.3) | 0.004 |
| No hobbies, n (%) | 37 (11.4) | 31 (21.1) | 0.007 |
| Weakening of neighbor relations, n (%) | 53 (16.4) | 39 (26.5) | 0.01 |
| Weakening of human relations, n (%) | 39 (12.0) | 21 (14.3) | 0.55 |
| Falls, n (%) | 61 (18.9) | 32 (21.8) | 0.53 |
| No long-distance walking, n (%) | 114 (35.2) | 63 (42.9) | 0.12 |
| Visual disturbance, n (%) | 13 (4.0) | 9 (6.1) | 0.35 |
| Stumbling, n (%) | 46 (14.2) | 27 (18.4) | 0.27 |
| Fear of falling, n (%) | 6 (1.9) | 9 (6.1) | 0.02 |
| Recent hospitalization, n (%) | 41 (12.7) | 27 (18.4) | 0.12 |
| Appetite loss, n (%) | 10 (3.1) | 13 (8.8) | 0.01 |
| Chewing difficulty, n (%) | 24 (7.4) | 14 (9.5) | 0.47 |
| Weight reduction, n (%) | 28 (8.6) | 18 (12.2) | 0.24 |
| Muscle and fat wasting, n (%) | 71 (21.9) | 44 (29.9) | 0.07 |
| Hypertension, n (%) | 234 (72.2) | 109 (74.1) | 0.74 |
| Diabetes, n (%) | 92 (28.4) | 34 (23.1) | 0.26 |
| Dyslipidemia, n (%) | 102 (31.5) | 36 (24.5) | 0.13 |
| Ischemic heart disease, n (%) | 40 (12.4) | 26 (17.7) | 0.15 |
| Stroke, n (%) | 23 (7.1) | 13 (8.8) | 0.58 |
| Chronic kidney disease |  |  |  |
| G1, n (%) | 7 (2.2) | 7 (4.8) | 0.25 |
| G2, n (%) | 161 (49.7) | 76 (51.7) |  |
| G3, n (%) | 152 (46.9) | 64 (43.5) |  |
| G4–5, n (%) | 4 (1.2) | 0 (0.0) |  |

Table S5. Symptoms of insomnia and sleep duration

|  | < 6 hours | 6–7 hours | 7–8 hours | 8–9 hours | ≥ 9 hours | *P* value |
| --- | --- | --- | --- | --- | --- | --- |
| N | 79 | 216 | 429 | 482 | 385 |  |
| DIS, n (%) | 24 (30.4) | 46 (21.3) | 103 (24.0) | 143 (29.7) | 115 (30.0) | 0.04 |
| DMS, n (%) | 22 (27.8) | 48 (22.2) | 129 (30.1) | 197 (40.9) | 195 (50.8) | < 0.001 |
| DIMS, n (%) | 38 (48.1) | 75 (34.7) | 200 (46.6) | 273 (56.6) | 257 (66.9) | < 0.001 |

*Abbreviations:* *DIS* difficulty initiating sleep; *DIMS* difficulty initiating and/or maintaining sleep; *DMS* difficulty maintaining sleep.
